# Structural brain basis of latent factors of executive functions in childhood

**DOI:** 10.1016/j.dcn.2024.101504

**Published:** 2024-12-28

**Authors:** Yongjing Li, Keertana Ganesan, Claire R. Smid, Abigail Thompson, Roser Cañigueral, Jessica Royer, Boris Bernhardt, Nikolaus Steinbeis

**Affiliations:** a Division of Psychology and Language Sciences, UCL, London WC1H 0AP, UK; b Clinical Psychology and Behavioral Neuroscience, Faculty of Psychology, Technische Universität Dresden, Dresden, Germany; c Department of Neurology and Neurosurgery, McGill University, Quebec, Canada

**Keywords:** Executive functions, Cognitive control, Latent factors, Childhood, Brain Structure

## Abstract

Executive functions can be classified into processes of inhibition, working memory and shifting, which together support flexible and goal-directed behaviour and are crucial for both current and later-life outcomes. A large body of literature has identified distinct brain regions critical to performing each of these functions. These findings are however predicated on a piecemeal and single-task approach. It is therefore unclear to what extent these associations reflect task-specific features or actual constructs of executive functions. Here, in a sample of 141 children aged 6–13 years, we administered a battery of 9 executive function tasks, derived latent factors of inhibition, working memory, and shifting and examined their associations with markers of brain structure (whole-brain cortical thickness). We identified associations between working memory and cortical thickness of right superior frontal and left medial temporal lobe as well as associations between shifting and cortical thickness in bilateral frontal and occipital lobes and left medial and anterior temporal lobes. While working memory and shifting shared a cortical substrate in right superior frontal cortex as well as left middle and inferior temporal regions no significant brain clusters were associated with inhibition. We discuss these findings in relation to theories of executive functions and their development.

## Highlights

• Explored links between latent factors of executive functions and brain structure.
• No associations between inhibition and brain structure.
• Shared substrate between memory and switching in right superior frontal regions.

## 1 Introduction

Accomplishing short- and long-term goals requires control of thoughts and actions. Executive functions (EFs) describe a cluster of cognitive processes that enable such goal-directed behaviour (Botvinick and Braver, 2015, Diamond, 2013a). Specifically, three core executive functions have been identified: stopping pre-potent responses and impulses (inhibition), retaining and manipulating goal-related information (working memory), and responding flexibly to changes in the environment (shifting). Impairments in executive functions have been associated with poor behavioural outcomes and clinically relevant behaviours of neurodevelopmental and externalising disorders (Mar et al., 2022, Wodka et al., 2007). In fact, it has been suggested that poor executive functions may constitute a transdiagnostic marker that cuts across multiple diagnoses of mental health problems (Caspi et al., 2014). Identifying the neural underpinnings of potentially clinically relevant markers may aid establishing diagnostic boundaries at multiple levels (Auerbach, 2022). However, efforts to advance this agenda have been hampered by a piecemeal approach, which precludes being able to say anything meaningful about executive functions beyond specific task performance. Here we overcome this by using multiple EF tasks, deriving latent EF factors and linking this with markers of brain structure, namely cortical thickness. We focus on middle to late childhood, a period that is marked by heightened neural and behavioral plasticity, and that at least in comparison to adolescence has received less attention but may be particularly important at least when thinking of and designing preventative interventions and before the onset of mental health problems.

Research indicates that EFs emerge as early as infancy and undergo protracted development into early adulthood (Diamond, 2013b, Wiebe and Karbach, 2017). Collectively, EFs mature rapidly in middle childhood, although there are differences in the rate of developmental progression between the three EF functions (Davidson et al., 2006, Xu et al., 2013). Similarly, the neural substrates reported to underpin EFs undergo protracted development (Fiske and Holmboe, 2019, Shanmugan and Satterthwaite, 2016). A core set of brain regions has been consistently implicated in executive functions in adults, comprising a so-called fronto-parietal network including dorsolateral prefrontal cortext (DLPFC), bilateral inferior frontal gyrus (IFG), inferior parietal lobule (IPL), superior parietal lobule (SPL) and pre-motor areas as well as a so-called cingulo-opercular network, comprising the dorsal anterior cingulate cortex (ACC) and the bilateral anterior insula (Dosenbach et al., 2008, Dosenbach et al., 2007). Comprehensive functional brain imaging studies in children (Engelhardt et al., 2019) as well as meta-analytic studies of brain activations during EF tasks in children (McKenna et al., 2017, Houde et al., 2010) report brain regions that are largely consistent with those identified in adults, including the bilateral prefrontal cortex, bilateral insula and left and right parietal regions. Further, extensive changes in frontal and parietal cortical volume and functional connectivity over development have been shown to mediate age-related improvements in executive functions (Buss and Spencer, 2018, Tamnes et al., 2010a).

A key question in the study of executive functions and its development is whether each are indeed separable constructs or whether they effectively represent different manifestations of the same underlying process (Fiske and Holmboe, 2019, Friedman and Miyake, 2017, Niendam et al., 2012). Also known as the diversity-unity debate, developmental studies suggest a unified structure of EFs in childhood, which supposedly matures into distinct subprocesses (Brydges et al., 2014, Wiebe et al., 2008). Neuroimaging studies have been used to arbitrate between these distinct positions (Engelhardt et al., 2019, McKenna et al., 2017), with distinct brain regions supporting EF processes being taken as evidence in favour of separate processes. The relevance of whether or not EFs are underpinned by distinct or common processes is also apparent in the design of interventions aiming to improve EFs (Ganesan et al., 2024, Steinbeis, 2023), since it has been argued that targeting common processes might lead to wider benefits (Wass et al., 2012), particularly in relation to development, where brain regions become increasingly specialised (Johnson, 2011). To date a plethora of studies has examined the functional and structural underpinnings of separate EFs within the same populations, finding distinct yet overlapping neural substrates that underlie EF functions (McKenna et al., 2017, Collette et al., 2006). Specifically, in adults the bilateral frontal-parietal network (FPN) has been found to underpin all three functions and proposed to support processes shared by all EFs (McKenna et al., 2017, Cole et al., 2013). A recent meta-analysis of fMRI studies of EF in children and adolescents identified a set of clusters shared between all three EFs comprising right and left supplementary motor area, left and right middle cingulum, and left and right superior and middle frontal gyri (McKenna et al., 2017). Equally, a host of subcortical as well as frontal and parietal cortical regions have been reported to represent processes distinct to each EF (Fiske and Holmboe, 2019, McKenna et al., 2017, Tamnes et al., 2010a, Aron, 2007, Crone and Steinbeis, 2017) (e.g. inferior frontal gyrus uniquely supports response inhibition (Aron et al., 2007); Swick et al., 2008). Unique brain-EF associations have been less robust in developmental populations however, particularly in children (McKenna et al., 2017). Overall, there are only very few studies that have investigated this question in developmental populations using appropriate designs (i.e. meta-analyses of multiple studies; within-participant studies of brain associations with multiple measures).

We addressed the question of shared and unique neural substrates of EF processes by focussing on cortical thickness. While brain function and structure are evidently related (Steinbeis et al., 2012), cortical thickness may be better suited to study the neural underpinnings of individual differences given its high test-retest reliability (Hedges et al., 2022), which is higher than typical for functional measures (Elliott et al., 2020, Noble et al., 2019). Prior work has identified age-dependent and -independent associations between EFs and cortical thickness in prefrontal and parietal regions during child and adolescent development (Tamnes et al., 2010b, Brito et al., 2017). The patterns of these relationships however have also been variable, with some studies showing cortical thinning to relate to improved executive functions (Tamnes et al., 2010b, Squeglia et al., 2013, Kharitonova et al., 2013) and others showing the reverse (Schnack et al., 2015). This picture is also somewhat complicated by typical cortical thinning across the cortex from childhood to adulthood (Bethlehem et al., 2022, Wierenga et al., 2014). One issue with the measurement of EFs and particularly their associations with neural mechanisms, structural or functional is that most studies employ single measures of executive functions (Tamnes et al., 2010a, Snyder et al., 2015). It is well known that executive function tasks are rarely pure, in terms of capturing single sub-processes that would map discretely onto specific neural substrates (Friedman and Banich, 2019, Enkavi et al., 2019). For instance, while inhibition may be the core process in classic inhibition tasks such as the stop signal task, it will also require monitoring and sustained attention (Chatham et al., 2012), working memory to recall task instructions as well as shifting abilities to alternate between sets of responses. Such task impurity suggests that associated neural correlates may not represent any specific EF process, but presumably a combination of processes or indeed very specific task demands (Snyder et al., 2015). These issues may contribute to the inconsistency in neural correlates observed between studies (McKenna et al., 2017, Tamnes et al., 2010a). One solution to ameliorating the task-impurity problem is to use a multi-measurement approach of multiple tasks of each EF (Kane and Engle, 2003, Smid et al., 2021) may be best positioned to identify the neural underpinnings of EF functions and to assess their relationship with one another. The use of more than one task to measure any of the three given EF allows deriving factors, which in turn are more likely to reflect each EF process without extraneous and specific task demands. Here we applied a factor approach to performance on nine EF tasks in a moderately sized cohort of children (*N* = 148) aged 6–13 years to measure task-independent associations between executive functions and brain structure and thus put the unity-diversity hypothesis to a rigorous empirical test. Given the age range we examine both age-dependent and age-independent associations between executive functions and cortical thickness.

We first look at associations between cortical thickness and each individual EF and then investigate the overlap between these. In line with robust associations between EF and frontal and parietal brain regions across multiple modalities we expected to replicate these relationships with the present analytic approach. Given the theoretical framework of increased functional specialisation with age (Johnson, 2011, Johnson, 2001) as well as the absence of unique associations between specific EFs and functional activation in prior meta-analyses (McKenna et al., 2017), we predicted substantial overlap in the association of cortical thickness and different executive functions in both frontal and parietal brain regions, indicating support for a unified set of brain regions to underpin EFs.

## 2 Materials and methods

### 2.1 Participants

A total of 262 typically developing children were recruited for the study (6.03–13.31 years; Age M = 8.97 years, Females = 52.84 %) from schools within Greater London in the United Kingdom (data collection started in Mar 2019 and ended in Mar 2021). The UCL ethics committee approved the study (Protocol number: 12271/001). In accordance with this, written consent was obtained from both parents and children after providing a description of the study. Socioeconomic status was assessed based on employment and education of both parents (Supplementary Materials Table 1). Subjects were excluded based on prior history of learning disabilities. Further, a safety protocol was followed for the scanning session that excluded some children (e.g. metal in the body; claustrophobia). A successful anatomical scan was collected from a subset of 141 participants (6.06–13.3 years, Age M = 9.12, Females = 55.3 %). Within our final sample there was a positive skew in SES (M = 1.64; on a scale of 1–5, where 1 is the highest score attainable). Ethnic composition of the subset sample was as follows: Asian = 12.59 %; Black = 3.70 %; Mixed/multiple ethnic groups = 17.78 %; White = 64.44 %; Other = 1.48 %.

**Table 1:** Correlations between age and EF abilities.

Measures	Age	Inhibition	Shifting
Age	1.00	-	-
Inhibition	0.42	1.00	-
Shifting	0.52	0.57	1.00
Working Memory	0.52	0.85	0.90

Note: All observed correlations were significant at *p* < .001.

### 2.2 Executive function tasks

A total of 9 executive function tasks were collected, each with an emphasis on assessing a distinct executive function (i.e. inhibition, working memory and shifting). Visual designs of these tasks have been shown in Supplementary Figures S1–8. For all tasks, participants were presented with practice trials before main trials were administered, where they had to attain a criterion threshold for accuracy. Additionally, comprehension questions were employed to ensure participants understood the rules for each task. Rules were repeated if participants answered incorrectly on any of the questions. The experimenter noted if the participant still failed to comprehend the task. All participants managed to pass these comprehension questions; therefore, no individual was excluded from the analysis.

#### 2.2.1 Inhibition tasks

##### 2.2.1.1 Stop-signal reaction time task

A measure of response inhibition was administered via a child-friendly version of the SSRT (Matzke et al., 2018). Ten practice trials were administered before 80 trials of the main task. Each trial started with the presentation of a fixation cross of 1250 ms. During the task, participants were asked to press the left arrow key when seeing a ‘go’ signal (i.e. a honey pot) on the left side of the screen and the down arrow key when the signal appeared on the right side. On 25 % of the trials (i.e. a ‘stop’ trial), a picture of bees was presented after the honey pot. This served as the ‘stop’ signal. The stop signal delay (SSD) started at 200 ms, decreased by 50 ms after a successful ‘stop’ trial, and increased by 50 ms after an unsuccessful ‘stop’ trial. As a measure of inhibition, a mean SSRT was calculated using the integration method (Verbruggen et al., 2019). Several studies have validated the SSRT as a measure of response inhibition (Logan et al., 2014) and it is correlated with self-report measures of impulsive behaviours in young adults (Logan, 1997).

##### 2.2.1.2 Flanker inhibition task

Participants completed a child-friendly version of the Eriksen Flanker inhibition task (Eriksen and Eriksen, 1974). Children were presented with a row of fish on the screen. They were required to focus on the fish in the centre (named Chloe) and indicate the direction in which it was swimming (i.e. left key response required when the fish was facing left; down key response required when the fish was facing right). Participants were told to ignore the direction other fish swim in and only indicate the direction Chloe swam in. On congruent trials, all fish faced the same direction. On incongruent trials, surrounding fish faced the opposite direction to Chloe. Fish were presented for 700 ms before they disappeared. Participants were given a maximum of 2500 ms to respond from stimulus onset. A total of 20 congruent trials and 20 incongruent trials were administered. This task was previously validated as suited for children of the present age range (McDermott et al., 2007, Mullane et al., 2009). The difference in both reaction times and error rates between incongruent trials and congruent trials was calculated.

##### 2.2.1.3 Stroop task

Participants completed a child-friendly version of the Stroop task (Williams et al., 2007). The task was introduced as the ‘Farm Animal’ game, where they were told to match animals to their homes (e.g. dog to a kennel). They were presented with both auditory stimuli of an animal sound (e.g. ‘bark’, ‘meow’, ‘croak’ for a dog, cat, and frog, respectively) and visual stimuli of the animals. Crucially, participants were asked to match animals to where they live (e.g. frog to a pond). They were told to listen carefully to an auditory cue indicating the animal type (e.g. frog – ‘ribbit’) and not to pay attention to the visual cue of the animal presented on the screen. Trials lasted for 10,000 ms within which participants had to make a response. While audio stimuli was presented for 600 ms, visual stimuli was presented until participants made a response (max of 1000 ms). A blank screen with a ‘cross’ was presented between trials for 10,000 ms (ITI). On congruent trials, both auditory and visual cues matched (e.g. frog presented on screen and ‘ribbit’ tone played). On incongruent trials, auditory and visual cues did not match (i.e. dog presented on screen and ‘ribbit’ tone played). Participants completed 72 trials in total, with 36 congruent and 36 incongruent trials. The differences in both reaction times and error rates between incongruent trials and congruent trials were calculated.

#### 2.2.2 Working memory tasks

##### 2.2.2.1 N-back task

Both a 1-back and 2-back versions were administered to measure working memory (Chen et al., 2008). The task was adapted to be child-friendly and introduced as the ‘Dino-Donut’ game, where participants were told that dinosaurs were lining up to eat some donuts. For the 1-back task, they were told to stop dinosaurs that tried to eat a donut twice in a row and to press the spacebar if they appeared consecutively to stop them. For the 2-back task, they were told that the dinosaurs became sneakier, and this time they should press the spacebar if the same dinosaur appeared two trials prior. Stimuli were shown for 500 ms followed by a 1500 ms Inter-Stimulus-Interval (ISI). Responses had to be made before the onset of the next stimulus presentation. Participants completed 80 trials in total, 40 for each n-back condition. A d-prime score based on hit rate and false alarm rate was calculated for both 1-back and 2-back tasks.

*Corsi block-tapping task.* Working memory span was also assessed using a Corsi block-tapping task, which measures visuo-spatial working memory span with a higher value indicating a higher working memory span (Pagulayan et al., 2006). This task consisted of ‘Freddy the frog’ jumping between nine potential locations designed as lily pads. The participants followed the jumps by clicking on the lily pads in a forward sequence. Trials commenced with a count-down from three to one to alert participants to the start of a trial. Then the stimulus of the frog jumping was shown for 600 ms for every jump. The ISI was fixed to 600 ms Participants completed three practice trials with feedback and there was a total of 14 main trials. Initially, participants had to remember and click on two lily pads. The task employed an adaptive staircase design where the working memory load (i.e. number of lily pads to remember) increased by one when participants made two consecutive correct answers. The maximum working memory load attained was used as a working memory span measure.

#### 2.2.3 Shifting tasks

##### 2.2.3.1 Cognitive flexibility task

A child-friendly version of a cognitive flexibility task assessed participants' ability for rule switching across dimensions (using sound cues: ‘animal’ or ‘size’). If a sound cue of ‘animal’ was played, participants had to indicate if the animal was a cat or dog. If a sound cue of ‘size’ was played, participants had to indicate if the animal was big or small (Karbach and Kray, 2009). Participants had 10 seconds to respond before the trial timed out, during which the stimuli remained on the screen—responses made before 200 ms after stimulus onset were not recorded. The inter-trial interval (ITI) was jittered and ranged from 1000 ms to 1200 ms. Stay trials were preceded by a trial with the same rule (e.g. deciding on the type of animal was presented twice in a row). During switch trials, the current trial was preceded by a trial in a different dimension (i.e., participants had to first respond to the size of the animal and then to the type of animal that is presented). Following a practice block, participants completed 40 trials (consisting of 28 stay trials and 12 switch trials). Participants completed 20 single-dimension trials in two blocks and 40 mixed trials in one block. The difference in reaction times between switch trials and stay trials was calculated.

##### 2.2.3.2 Flanker shifting task

The participants completed a child-friendly version of the Eriksen Flanker shifting task (Karbach and Kray, 2009). Children were presented with a row of fish on the screen. They were told that all the fish swim in the same direction. However, that two colours of fish would appear: orange and purple fish. When orange fish were presented, they were instructed to indicate the direction in which the fish swam (i.e. left key response required when the fish faced left; down key response required when the fish faced right). When purple fish were presented, they were instructed to indicate the opposite direction in which the fish swam (i.e. left key response required when the fish was facing right; down key response required when the fish was facing left). Fish were presented for 700 ms before they disappeared. Participants were given a maximum of 2500 ms to respond from stimulus onset. Stay trials were defined as those where the rule for the previous trial was the same as the current trial (i.e. purple trial following a purple trial; orange trial following an orange trial). Switch trials were defined as those where a rule change has occurred (i.e. purple trial following an orange trial; orange trial following a purple trial). Based on this, there were 28 stay trials and 12 switch trials. The difference in both reaction times and error rates between switch trials and stay trials was calculated.

#### 2.2.4 Complex EF tasks

***AX-***CPT task*.* Reactive and proactive control were measured using a child-friendly version of the AX-CPT paradigm (Chatham et al., 2009). The task was introduced as the Fruit Island game. An ‘A’ or ‘B’ cue (i.e. dog or cat) was presented in the middle of the screen for 500 ms, followed by an inter-stimulus interval of 750 ms and then a probe ‘X’ or ‘Y’ (orange or apple) during which participants had to make their response. Participants were instructed to press the left key whenever an ‘X’ followed an ‘A’ (i.e. AX trials) and to press the down arrow key for all other cue-probe combinations. Importantly, they were instructed to only respond once the probe had been presented and were alerted of this if they made a response before the probe was presented. Participants had a maximum of 6000 ms to make a response. Responses were followed by an inter-trial interval of 1500 ms. Proportions of trial types were based on previous studies (Chatham et al., 2009, Richmond et al., 2015) where 40 % of trials were AX trials and all other trials (i.e. AY, BX, BY trials) were presented 20 % each. Trials were presented randomly. Ten practice trials were administered where feedback was provided, followed by 60 main trials.

### 2.3 Brain structure data acquisition

High-resolution T1-weighted images were obtained using a Siemens 3.0 Tesla Prisma scanner located at the Birkbeck-UCL Centre for Neuroimaging (BUCNI) equipped with a 32-channel whole-head coil. Images were acquired with the sequence tfl3d1_16ns with a flip angle of 9 Echo Time was set to 0.00298, and Repetition Time to 2.3. A total of 208 slices with a voxel size of 1x1x1 mm3 were collected, and the acquisition matrix ranged over 256 × 256. To limit head motion, children were requested to keep their heads as still as possible and foam inserts were placed between the head and head coil to ensure the head was snug in the coil. Visual stimuli were projected onto a screen in the magnet boar that could be viewed via a mirror attached to the head coil. Participants watched cartoons without sound during the acquisition of the structural scan.

### 2.4 Statistical analysis

#### 2.4.1 Executive function factors

**Fig. 1:**
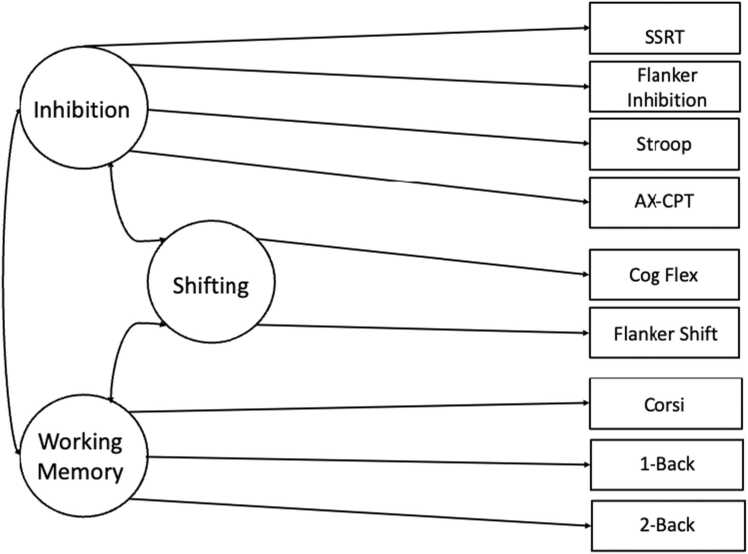
Loading of executive function tasks on inhibition, shifting and working memory factors. Inhibition factor is mainly correlated with participant’s performance in Stop Signal Response Task, Flanker Inhibition Task, Stroop Task and AX-CPT task; Shifting factor is mainly correlated with performance in Cognitive Flexibility Task and Flanker Shift Task; Working Memory factor is mainly correlated with performance in CORSI Task, and N-back Task. All three factors are sub-factors of Executive Function.

**Fig. 2:**
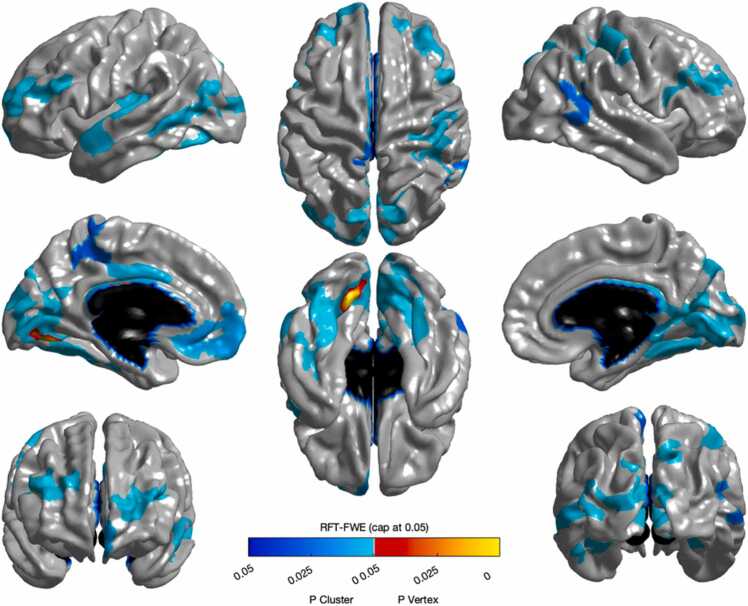
Significant reductions in cortical thickness with age in eleven clusters in bilateral temporal lobes, and right frontal lobe.

Outliers were removed from behavioural executive function measures by removing datapoints falling two standard deviations below or above the mean. Then, a confirmatory factor analysis (CFA) was performed using lavaan in Rstudio to create latent factors of EFs (Rosseel, 2012). FIML was used to deal with any missing data in the dataset. Several models including one-factor model and two-factor model (inhibition, shifting together with working memory as second factor) were fit, however, the model failed to converge for most models, with some of them displaying negative variances suggesting that models were mis-specified. Only two models converged: a model with a single factor encompassing all tasks and a model with three sub-factors of inhibition, shifting, and memory. There were no significant differences in model fits (Δ*χ*^2^ (3) = 1.69, *p* = .638). As individual task loadings for the unitary factor of EF was poor since the unitary factor of EF could be measured by multiple factors, we chose the model with the three sub-factors of executive functions for further analysis (Figure 2–1). This model was a good fit for our data (CFI/TFI > 0.95; RMSEA <.05). For each individual, a value of EF abilities was extracted from the fit model for further analysis. Specifically, values of inhibition, shifting and working memory abilities were extracted with larger values indicating better EF abilities.

#### 2.4.2 Cortical thickness

After converting the Dicom files to Nifti using dcm2niix, structural MRI images were processed with FreeSurfer (Fischl et al., 2002) (Version 6.0.0; http://surfer.nmr.mgh.harvard.ed) to label and segment cortex and white matter. All scans were visually inspected for quality, and if necessary, segmentation was manually corrected in FreeSurfer. All scans went through two rounds of inspections during which four independent inspectors conducted these checks, and one final inspector performed a final inspection of all scans. After corrections, scans were re-segmented using FreeSurfer and were inspected. Scans were excluded from the final analysis if their quality was inadequate (N = 18; due to movement or poor segmentation). Based on this, data was available from 123 participants with sufficiently high data quality. After pre-processing, sulcal and gyral features across individual subjects were aligned by morphing each subject’s brain to an average spherical representation that accurately matches cortical thickness measurements across participants while minimizing metric distortion. A 10 mm Gaussian smoothing kernel was applied to data to reduce measurement noise but preserve the capacity for anatomical localizations (Bernhardt et al., 2014a, Lerch and Evans, 2005). Cortical thickness data were analyzed using the SurfStat toolbox for Matlab (Worsley et al., 2009) (https://www.math.mcgill.ca/keith/surfstat). Linear regression models were used to assess the effects of age and executive function factors on cortical thickness at each vertex. Findings from the surface-based analyses were controlled for multiple comparisons using random field theory (Steinbeis et al., 2012, Bernhardt et al., 2014a, Bernhardt et al., 2014b, Worsley et al., 2009). This reduced the chance of reporting a family-wise error (FWE). The threshold for significance was set to a stringent *p* < 0.05. The Desikan-Killiany atlas (Desikan et al., 2006) was used to label any observed significant cortical thickness correlates.

We assessed associations between cortical thickness and individual EF factors and report the findings below. To test for shared neural substrates between these different EF factors we also look at the overlap of associations. Significant overlap would indicate support for the unity hypothesis of EF.

## 3 Results

### 3.1 Correlations with age

All three executive function factors were correlated with age (*r* > .42, *p* < .001; Table 1). Specifically, age was associated with better executive function abilities.

### 3.2 Cortical thickness and age

Several brain regions showed cortical thinning with age, including clusters in the bilateral temporal lobes and right frontal lobe (Fig. 2). For more details on these regions, see Supplemental Materials 1.

### 3.3 Inhibition

There were no significant clusters associated with inhibition.

### 3.4 Working memory

**Fig. 3:**
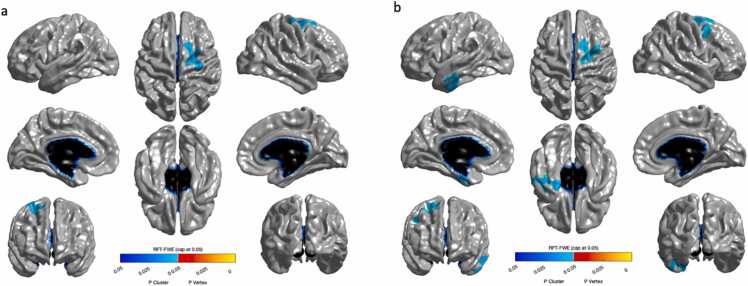
a) Significant associations between cortical thickness in one cluster in the right frontal lobe and working memory was found. b) After controlling for age, significant associations between cortical thickness in the right frontal lobe and the left temporal lobe were observed.

Working memory was positively associated with cortical thickness of one cluster in the right frontal lobe (Fig. 3a). This association was still significant after controlling for age. In addition, working memory was positively associated with cortical thickness of one cluster in the left temporal lobe (Fig. 3b; Figure S9).

### 3.5 Shifting

**Fig. 4:**
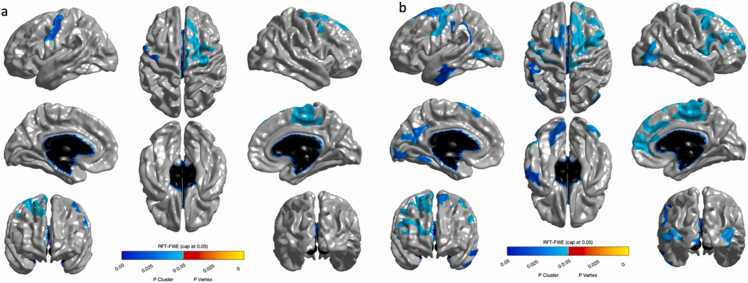
(a) Significant associations between cortical thickness in three clusters in the bilateral frontal lobe were observed. (b) After controlling for age, these associations remained and more extensive ones were found in bilateral frontal and occipital lobes and left medial and temporal lobes were observed.

Shifting was positively associated with cortical thickness in three clusters. Specifically, in the bilateral frontal lobes including the left and right precentral gyrus and right superior frontal gyrus (Fig. 4a). When controlling for age, shifting was positively associated with cortical thickness in eleven clusters (Fig. 4b; Figure S10). For more details on these regions, see Supplemental Materials 1.

### 3.6 Overlap between EF abilities

**Fig. 5:**
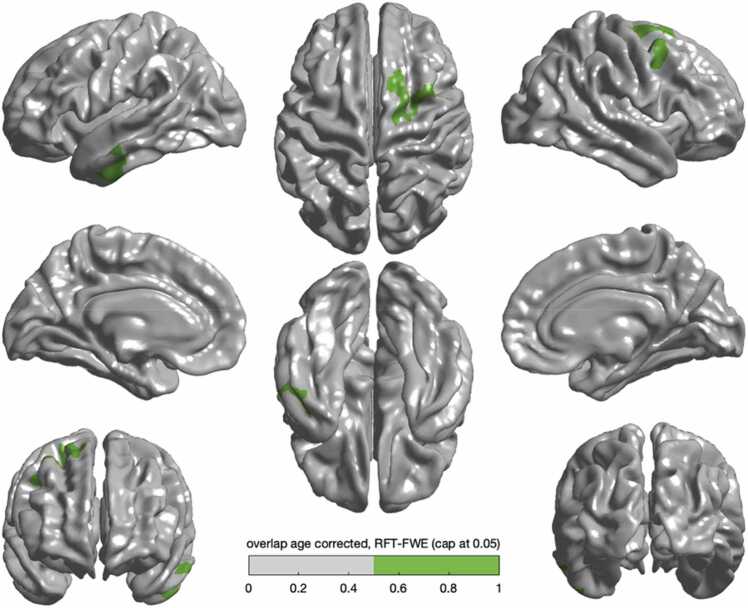
(a) Significant overlap in the right superior frontal and left temporal lobe was observed between working memory and shifting.

We visualised the overlap of associations between cortical thickness and working memory and shifting respectively. Both working memory and shifting were associated with cortical thickness in the right superior frontal area, right precentral area, right caudal middle frontal area and left inferior and middle temporal area. (Fig. 5; see Supplemental Materials 2).

## 4 Discussion

The study of executive functions and its supporting functional and structural neural architecture has long been dominated by a piece-meal approach, leaving it open whether associations relate to features specific to single tasks or underlying cognitive processes. We used a multi-measurement approach to capture EF processes above and beyond specific tasks, and sought to uncover associations between these processes and brain structure. We found large and widespread age-related thinning across frontal, temporal and parietal regions, both lateral and midline. We observed positive associations between working memory and cortical thickness and right superior frontal and left medial temporal lobe as well as positive associations between shifting and cortical thickness in bilateral frontal and occipital lobes and left medial and anterior temporal lobes. We observed no associations between inhibition and cortical thickness in any brain region and a notable absence of involvement of parietal brain regions across the board. Finally, working memory and shifting shared a cortical substrate in right superior frontal cortex as well as left middle and inferior temporal regions.

Using a multi-measurement approach of 9 experimental tasks in 141 children between the ages of 6–13 years we derived a 3-factor structure that fit our data, representing inhibition, working memory and shifting abilities (Völter et al., 2022). This allowed examining the structural brain basis of canonical executive function processes above and beyond specific task-related variance. In line with previous research, age was significantly correlated with all three executive functions (Garon et al., 2014) and functions were highly correlated with each other. No clusters were identified to be associated with the inhibition factor. This suggests that inhibition is not uniquely associated with any neural substrates and that any previously identified neural correlates found to specifically underlie inhibition may have represented task-specific elements or other EFs rather than inhibition per se (McKenna et al., 2017, Tamnes et al., 2010a, He et al., 2021, Saylik et al., 2022). Indeed, a meta-analysis found that activation in regions associated with inhibition, completely overlapped with common executive function areas (McKenna et al., 2017). It has further been argued that inhibition abilities may simply represent a generalised processing ability to support simpler goals in younger children with distinct executive processes developing in later childhood (Davidson et al., 2006, Friedman and Miyake, 2017, Miyake and Friedman, 2012a, Friedman et al., 2011, Friedman et al., 2008).

Working memory abilities were associated with cortical thickness of clusters in the right superior frontal and medial temporal lobe. This is in line with the literature suggesting frontal regions and the entorhinal cortex to play a crucial role in memory (McKenna et al., 2017, Takehara-Nishiuchi, 2014). Shifting was associated with cortical thickness in an extensive network of regions including bilateral frontal and occipital lobes and left medial and anterior temporal lobes. Shifting abilities have been argued to be more complex than other EFs, requiring attentional flexibility, monitoring of different task demands (Dajani and Uddin, 2015). This may explain why individual differences in cortical thickness of these multiple regions may be crucial in supporting the different processes involved in shifting abilities. Crucially, we identified a partial overlap in clusters associated with working memory and shifting, namely in the superior part of the right frontal lobe as well as left medial temporal lobe, pointing to a shared neural substrate underlying both abilities. This is in line with previous literature demonstrating common executive components underlying working memory and shifting (Engelhardt et al., 2019, McKenna et al., 2017), where these areas have been implicated in top-down processes such as planning and information selection (i.e. core components in EFs) functioning a ‘global’ executive network (Rodríguez-Nieto et al., 2022). Here we show that such shared processing becomes manifest in common associations with brain structure. In terms of the unity-diversity debate these findings paint an interesting picture, whereby a common set of superior frontal and temporal regions subserves both working memory and shifting, while an additional and extensive network of lateral and medial prefrontal and occipital regions is associated with shifting only. Thus frontal cortices appear to underlie common executive functioning, distinct brain cortices are selectively recruited for specific functions, namely shifting (Engelhardt et al., 2019, McKenna et al., 2017). Taken together, these findings support a theory of unity and diversity of executive functions during child development (Davidson et al., 2006, Friedman and Miyake, 2017, Miyake and Friedman, 2012a, Friedman et al., 2011, Friedman et al., 2008). We also note that associations found between EFs and cortical thickness involved predominantly frontal brain regions while parietal brain regions were not involved. While we did not test for the absence of an association with parietal cortices, we interpret this patterns as one of an increased specialised role of fronto-parietal regions in executive functions, whereby frontal regions appear present during childhood and across two of three core EF processes, and parietal regions assume their functional importance later.

We note some limitations of the present study. Our findings are restricted to middle childhood. Executive functions do not have the same developmental trajectories and progressions. While inhibition abilities have been observed in children as young as four-years old, shifting abilities continue to develop in adolescence (Davidson et al., 2006). Therefore, in particular, age-independent associations should be interpreted with this developmental population in mind. Further, we used cross-sectional data to examine neural correlates. Consequently, we are only able to interpret our findings as brain-behaviour associations (i.e. looking at underpinnings of executive functions). A longitudinal design would have allowed us to use mediation models to examine how brain maturation could explain increases in abilities.

In sum, using a multi-measurement approach, we sought to clarify the structural substrates of inhibition, working memory and shifting abilities. While there were no association with inhibition, we show a combination of frontal and temporal regions to underpin both working memory and shifting and a wider set of regions to be uniquely associated with shifting only. These findings suggest both unity and diversity in the neural underpinnings of EFs during development.

## CRediT authorship contribution statement

**Jessica Royer:** Formal analysis. **Nikolaus Steinbeis:** Writing – review & editing, Writing – original draft, Supervision, Project administration, Funding acquisition, Conceptualization. **Keertana Ganesan:** Writing – review & editing, Writing – original draft, Investigation, Formal analysis, Data curation, Conceptualization. **Claire R. Smid:** Writing – review & editing, Investigation, Formal analysis, Data curation, Conceptualization. **Abigail Thompson:** Writing – review & editing, Project administration, Data curation, Conceptualization. **Roser Cañigueral:** Writing – review & editing, Project administration, Investigation, Data curation, Conceptualization. **Yongjing Li:** Writing – review & editing, Data curation. **Boris Bernhardt:** Formal analysis.

## Declaration of Competing Interest

The authors declare the following financial interests/personal relationships which may be considered as potential competing interests: Nikolaus Steinbeis reports financial support was provided by European Research Council. Nikolaus Steinbeis reports financial support was provided by Economic and Social Research Council. Nikolaus Steinbeis reports financial support was provided by Jacobs Foundation. If there are other authors, they declare that they have no known competing financial interests or personal relationships that could have appeared to influence the work reported in this paper.

## Data Availability

Data will be made available on request.

## Footnotes

Appendix A Supplementary data associated with this article can be found in the online version at doi:10.1016/j.dcn.2024.101504.
