# Supplementary material for: Structural brain basis of latent factors of executive functions in childhood
Source: Dev Cogn Neurosci. 2024 Dec 28;71:101504. doi: 10.1016/j.dcn.2024.101504 (PMC11780108; doi:10.1016/j.dcn.2024.101504)
Supplement: Supplementary file 1 — Supplementary material [file mmc1.docx]

| **Supplementary Table 1.** Peak coordinates for all cluster associations have been shown. Brain areas are listed using DK atlas regions. | | | | | | |
| --- | --- | --- | --- | --- | --- | --- |
| **Measures** | **Association** | | **Coordinates (MNI)** | | | **Area** |
|  |  |  | **x** | **y** | **z** |  |
| Age | - | | 20 | -76 | 47 | Right lingual and fusiform |
|  |  | | -38 | -52 | -19 | Left lingual and fusiform |
|  |  |  | 48 | -21 | 43 | Right postcentral |
|  |  |  | -8 | 66 | 17 | Left rostral middle frontal |
|  |  |  | -36 | 1 | 15 | Left superior temporal |
|  |  |  | 38 | 36 | 33 | Right rostral middle frontal |
|  |  |  | -49 | -63 | 10 | Left middle temporal |
|  |  |  | -4 | -20 | 43 | Left posterior cingulate |
|  |  |  | -8 | 53 | -3 | Left medial orbital frontal |
|  |  |  | 60 | -42 | 2 | Right inferior parietal |
|  |  | | -7 | -51 | 41 | Left paracentral |
| Working Memory | + | | 21 | -4 | 61 | Right precentral |
|  |  |  |  |  |  |  |
| Working Memory  (age-corrected) | + | | 21 | -4 | 61 | Right precentral |
|  |  |  | -28 | -3 | -34 | Left middle temporal |
|  |  |  |  |  |  |  |
| Shifting | + | | 10 | 43 | 49 | Right precentral |
|  |  |  | -31 | -18 | 45 | Left precentral |
|  |  |  | 8 | -9 | 51 | Right superior frontal |
| Shifting (age-corrected) | + | | 8 | -9 | 51 | Right superior frontal |
|  |  |  | 20 | 62 | 16 | Right rostral middle frontal |
|  |  |  | 46 | 6 | 21 | Right parsopercularis |
|  |  |  | -31 | -18 | 45 | Left precentral |
|  |  |  | 47 | -74 | 12 | Right lateral occipital |
|  |  |  | -43 | -75 | 6 | Left lateral occipital |
|  |  |  | -18 | 4 | 70 | Left superior frontal |
|  |  |  | -2 | -62 | 31 | Left precuneus |
|  |  |  | -17 | -82 | 7 | Left lingual |
|  |  |  | -56 | -28 | 38 | Left supramarginal |
|  |  |  | -53 | -14 | -29 | Left middle temporal |

**Supplementary** **Table** **2**. Overlapping regions in cortical thickness between Working Memory and Shifting.

| Overlapping area | Percent |
| --- | --- |
| Right superior frontal area | 26.46% |
| Right precentral area | 20.18% |
| Right caudal middle frontal area | 26.91% |
| Left inferior temporal area | 12.11% |
| Left middle temporal area | 13.90% |
| Left superior temporal area | 0.45% |


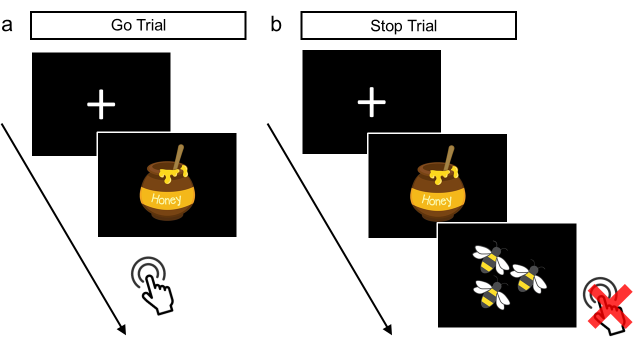


Figure S-1. Stop Signal Reaction Time (SSRT) task where on ‘go’ trials (a, participants were to make a response as fast as they could. On ‘stop’ trials where the ‘stop’ signal was presented (i.e. bees) participants were instructed to make no response.


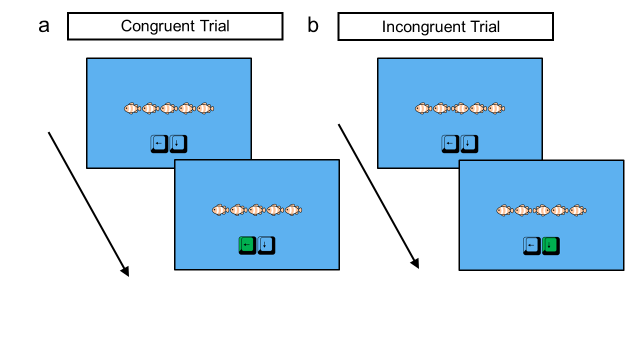


Figure S-2. Flanker inhibition task where participants had to indicate the direction of the middle fish (i.e. central target). During congruent trials (a), the central target stimulus faced the same direction as the other stimuli. During incongruent trials (b), the central target stimulus faced the opposite direction to the other stimuli.


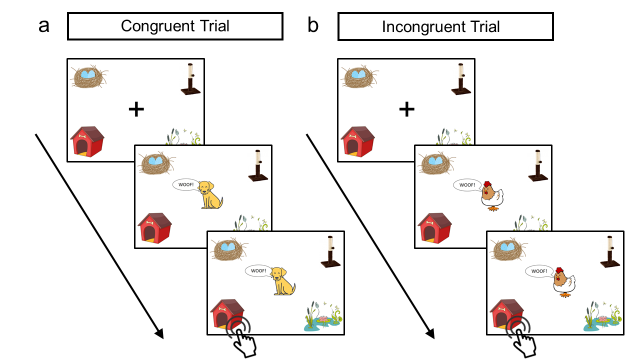


Figure S-3. Animal Stroop task where participants were told match the animal (based on auditory stimuli) to its home. On congruent trials (a) the visual stimuli of the animal matched the auditory stimuli. On incongruent trials (b) the visual and auditory stimuli did not match.


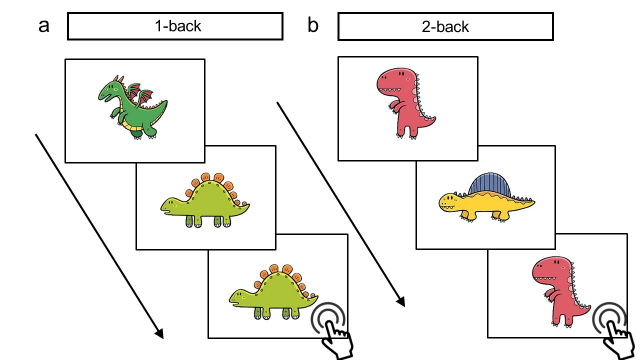


Figure S-4. N-back task where participants completed two variations (1- and 2-back). During the 1-back task, participants had to respond by pressing the spacebar if they saw the same dinosaur twice in a row. During the 2-back block (b), participants had to respond by pressing the spacebar if the current dinosaur was the same as two stimuli previously.


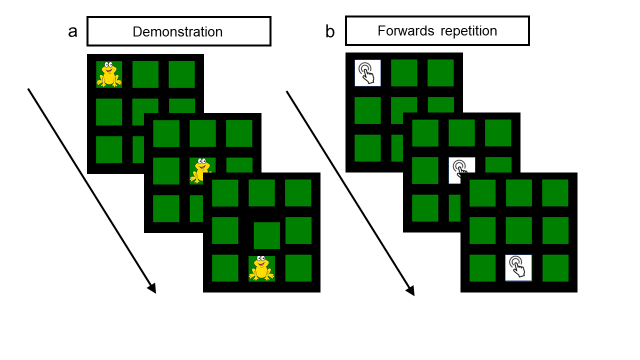


Figure S-5. Corsi block tapping task where on each trial (a), participants first observed the target stimulus ‘jumping’ between lily pads. Then participants were required (b) to repeat the sequence of ‘jumping’ by clicking on the correct lily pads.


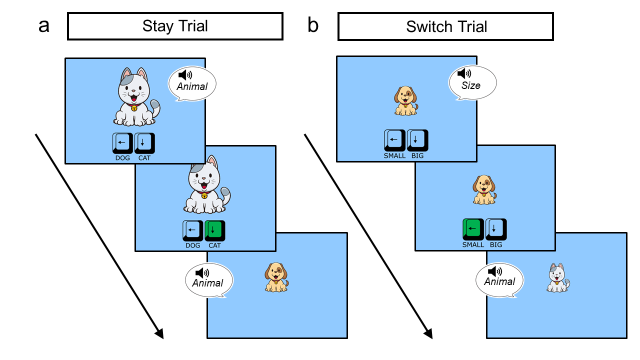


Figure S-6. Cognitive flexibility task where participants had to indicate either the type or size of animal presented. Stay trials were preceded (a) by a trial with the same rule. During switch trials (b), the current trial was preceded by a trial in a different dimension.


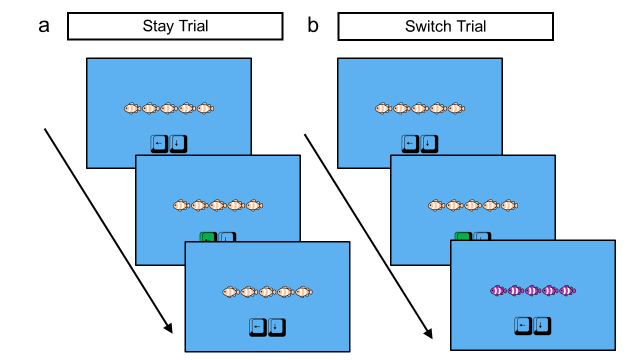


Figure S-7. Flanker shifting task where there were two different sets of rules (i.e. purple/orange). When orange fish were presented, they were instructed to indicate the direction in which the fish swam. When purple fish were presented, they were instructed to indicate the opposite direction in which the fish swam. (a) Stay trials were defined as those where the rule for the previous trial was the same as the current trial. (b) Switch trials were defined as those where a rule change has occurred.


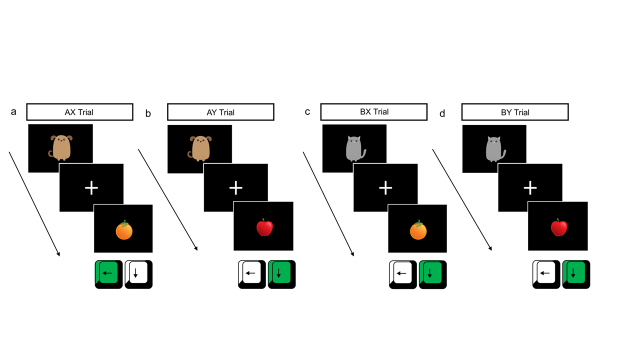


Figure S-8. AX-CPT task indicating proactive and reactive control. During AX trials (a), participants had to respond by pressing the left arrow key. In contrast, during all other trials (b-d) participants had to respond by pressing the down arrow key.


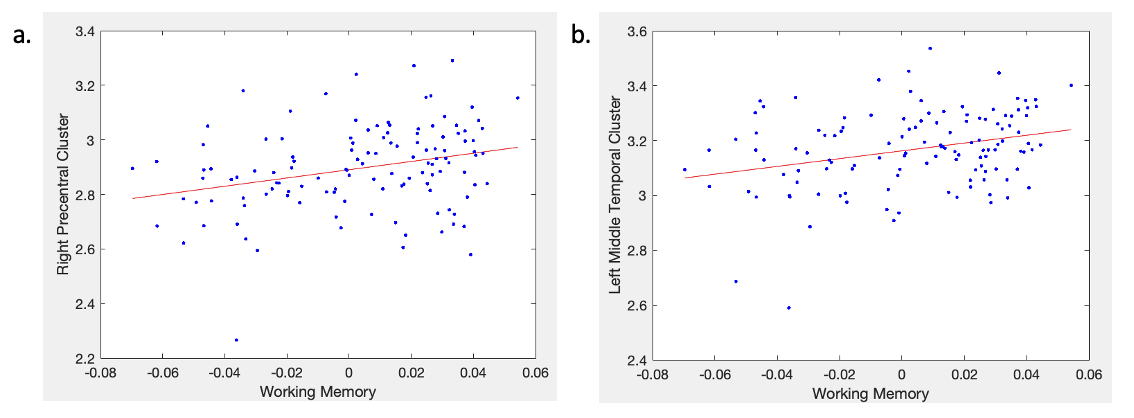


Figure S-9 Plots of ROI analysis for Cortical thickness of two significant clusters and working memory when controlling for age.


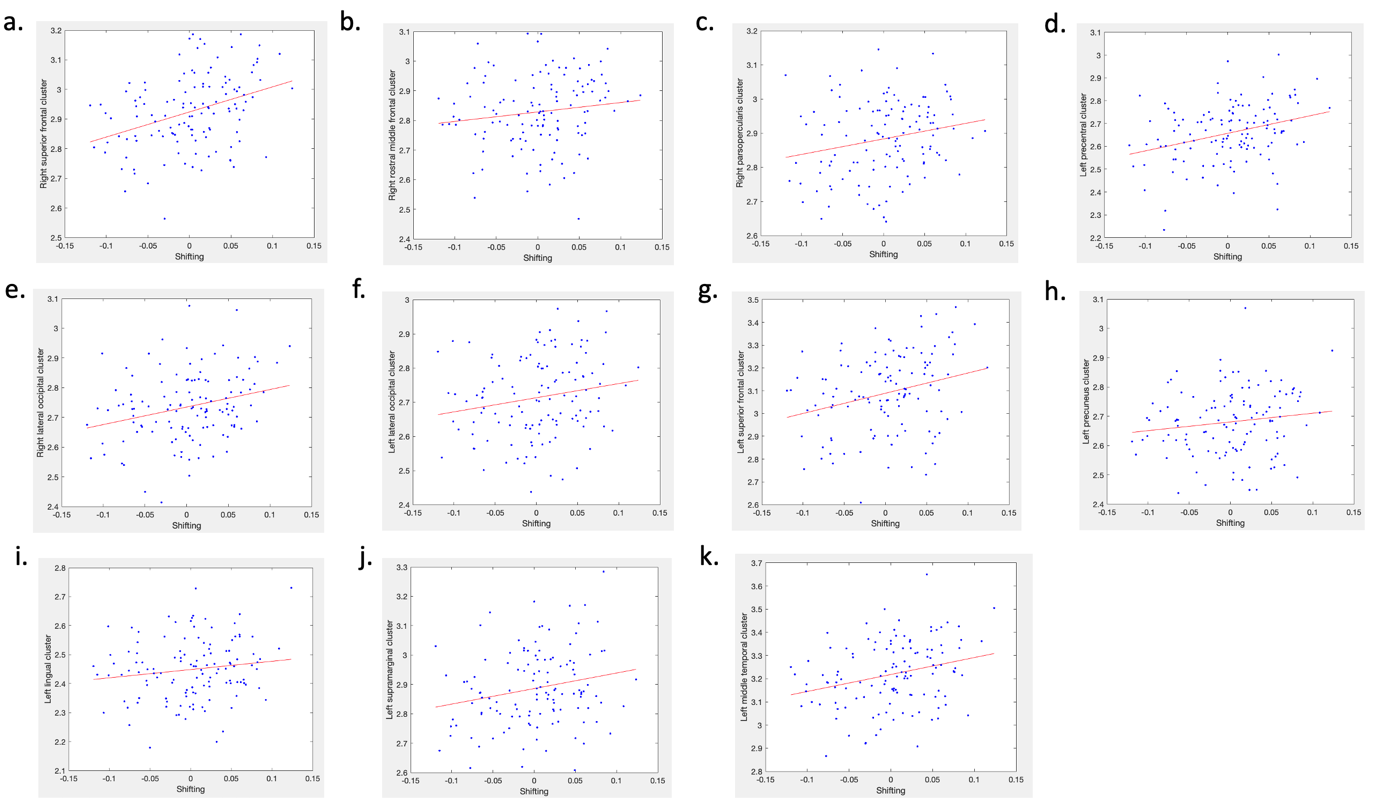


Figure S-10 Plots of ROI analyses for Cortical thickness of eleven significant clusters and reversed shifting value (higher shifting value, lower shifting ability) when controlling for age.
